# Locked the Car, Why Not the Computer: A Qualitative and Quantitative Study on Data Safety Compliance

**DOI:** 10.7759/cureus.17513

**Published:** 2021-08-27

**Authors:** Ghulam Dastagir Faisal Mohammed, Prakash Chandran, Zaina Mansoor, Momin Mohaddis

**Affiliations:** 1 Trauma and Orthopaedics, London Northwest NHS Trust, London, GBR; 2 Trauma and Orthopaedics, Warrington and Halton NHS Foundation Trust, Warrington, GBR; 3 Anaesthesia, Gandhi Hospital and Medical College, Hyderabad, IND; 4 Orthopaedics, Warrington Hospital, Warrington, GBR

**Keywords:** data safety, lock computer, cyber security, quality improvement projects, nhs

## Abstract

Information technology has become an integral part of health care in the United Kingdom National Health Service (NHS). All health care professionals are required to have a certain level of cyber ethics and knowledge of computers. This is assured by regular mandatory training. The government of the United Kingdom has charted out a course to strengthen cyber security and prevent any crises like Wannacry. Simple things like leaving a computer unlocked can pose a potential threat to the cyber security of the whole NHS. These cannot be addressed with money alone, as they involve complex interactions of human factors. Such seemingly simple non-compliance results often in harm to the patient or breach of confidentiality. We tried to find out the compliance among junior doctors to the Trust Information Technology (IT) Safe Usage Policy. We made interventions and interviewed junior doctors to find out the reasons for non-compliance. We re-audited in order to see if our interventions helped. We also audited compliance in another Trust independently, which showed that this problem is not specific to a particular trust. Here we suggest the changes that all Trusts can make and follow our model to audit their compliance.

## Introduction

Information technology in health care has become the solution for the housekeeping and information handling challenges a health care system is faced with today. It now plays a central role in health care delivery, which has now lead to a novel way of patient harm - via computer systems. A recent study found 2627 incidents of patient harm were recorded, three-quarters of which were preventable [1]. These failures were determined to be due to the complex interaction of three factors - machine-related, people-related, and environment-related [1]. The events related to patient harm due to information technology (IT) errors are frequently under-reported. There is very little data, which quantifies the true extent of this problem and the reasons behind it. More often than not, we see an unoccupied computer unlocked and the problems arising from this are common knowledge to all. They can range from a wrong request, exchange of notes to potentially breach of confidentiality and hacking. They can also lead to patient harm and death [2]. We set out to determine how compliant the junior doctors are with the above policy. The aim was to audit our compliance, investigate the causes preventing full compliance, and support the junior doctors by addressing these causes and improving compliance.

## Materials and methods

All unoccupied computers were checked in patient-accessible clinical areas (wards) of one wing in a busy District General Hospital in Cheshire County in the North-West of England. If an unoccupied computer was found the assessor would check if it is locked or not. If it was locked, we recorded whether more than one user was logged on. If it were not locked then the assessor would wait for three minutes at the computer to check if someone came around to own responsibility for the machine. If not, the computer ID, location, and type of programmes open were recorded on a Microsoft Excel sheet (Microsoft Corporation, Redmond, USA).

**Figure 1:**
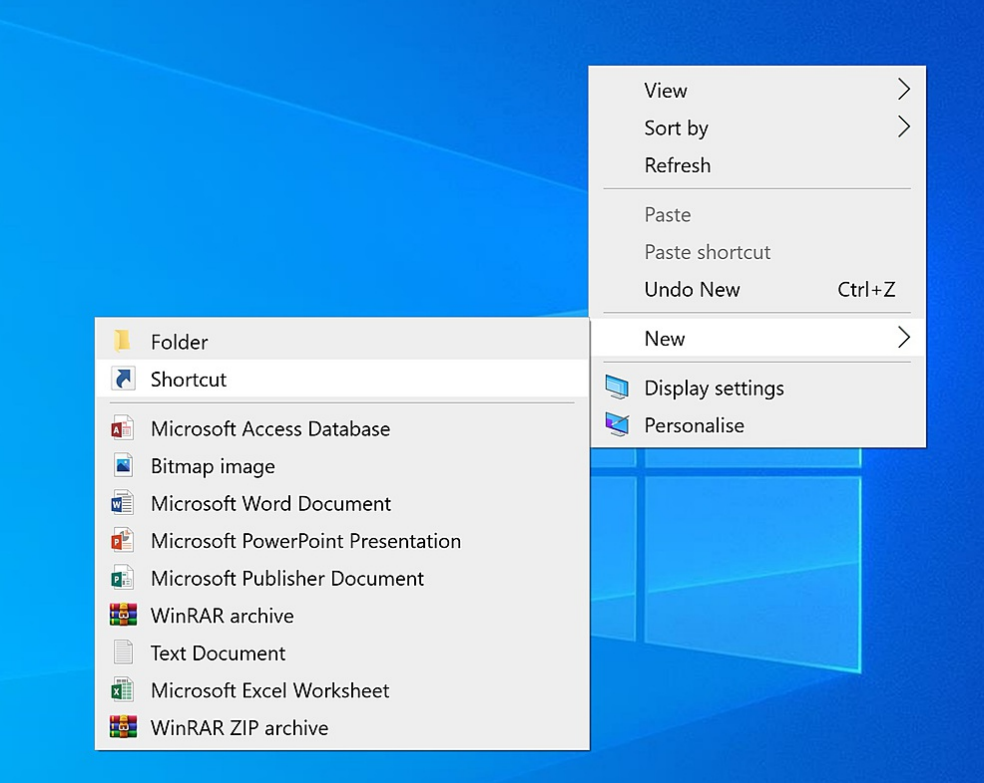
Right-click on the desktop to create a shortcut Hover the mouse pointer over "New" and then click on "Shortcut".

**Figure 2:**
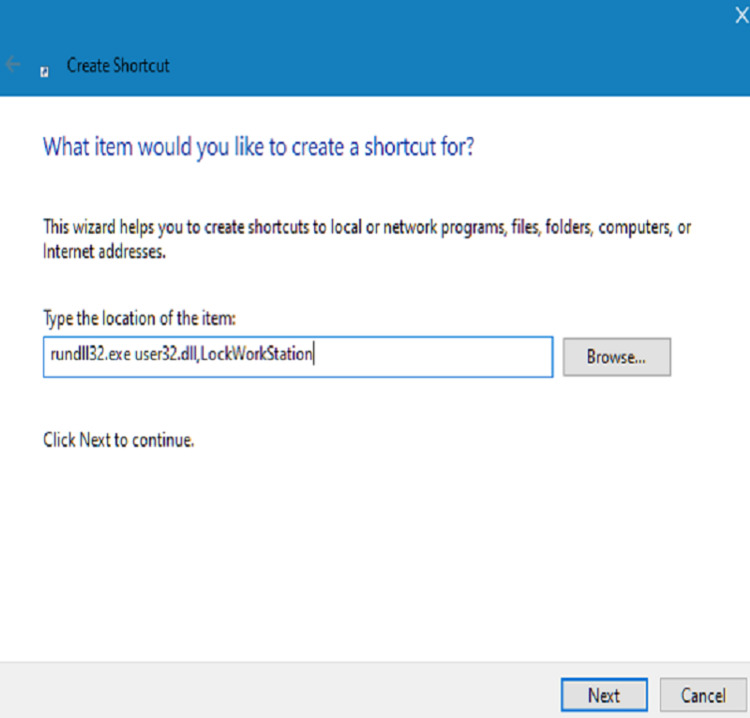
Wizard to create the shortcut The above dialogue box appears and type "rundll32.exe user32.dll,LockWorkStation" in the location address field as shown above and click "Next" to continue.

**Figure 3:**
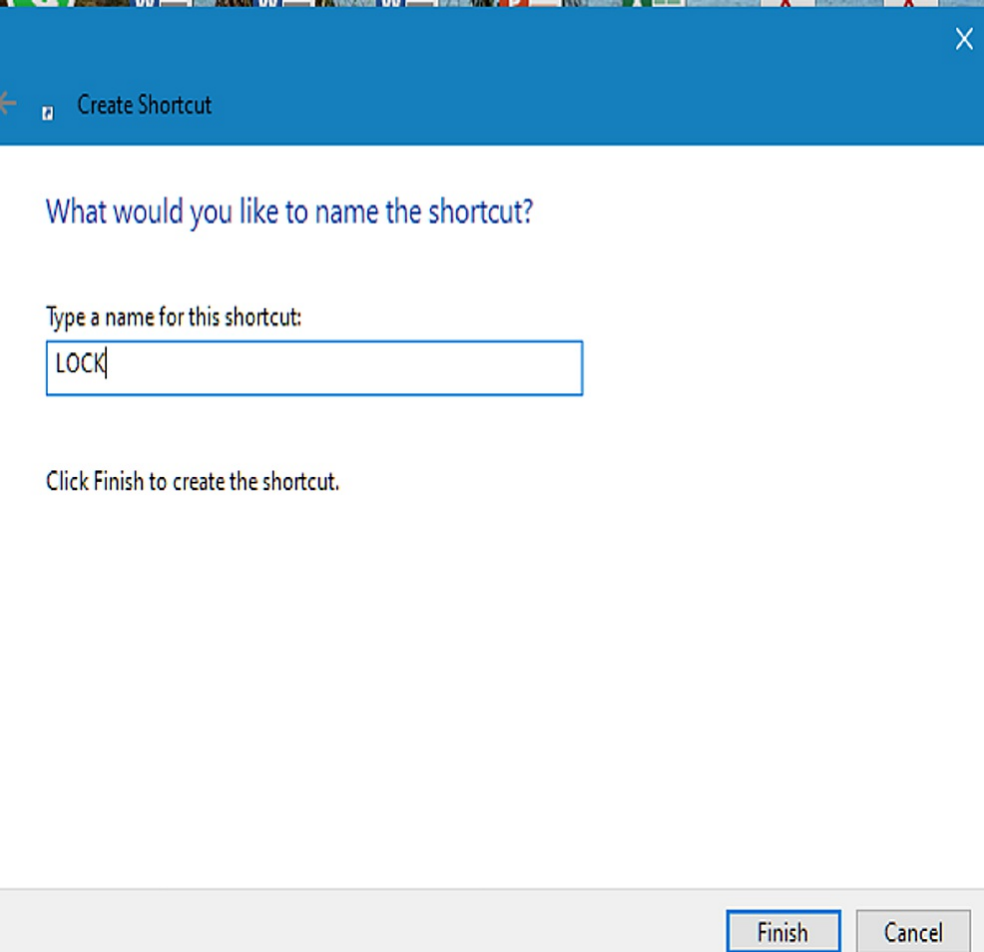
Name the shortcut Name the shortcut icon as you wish, for example, "LOCK", as shown above.

**Figure 4:**
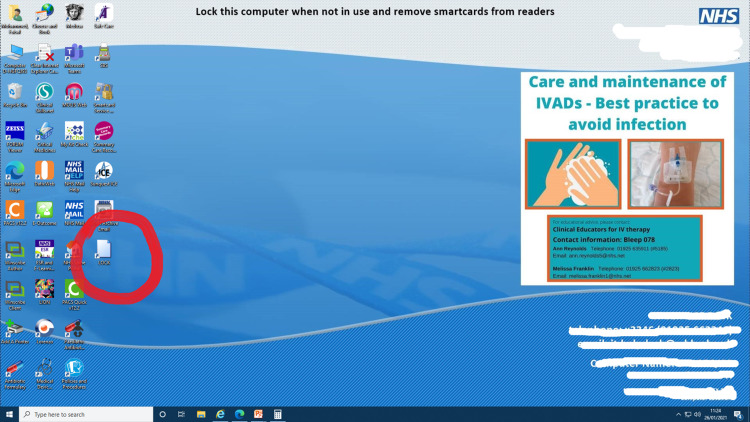
Shortcut on the desktop The shortcut you created appears on the desktop as shown. Double-click on it to lock the device instantly. Please also note at the top of the image, the reminder we displayed on the desktop screen on all Trust computers. All identifiable data have been blurred for security reasons.

We presented this audit and suggested interventions. We fed back to the IT department and requested them to consider altering the IT induction. We displayed a message on the computer wallpaper reminding people to log out. We sent a Trust-wide email communication informing people about the low compliance and reminding them to lock the computer when they leave the computer. We put up a message in the junior doctors' rooms, which read, “Locked the car? Why not the computer?” to increase compliance. We also interviewed 13 junior doctors across the medical and surgical departments to find out more about the reasons that would not allow a junior doctor to lock a computer when they were leaving it. We also presented easier ways of locking computers - 1. Using Ctrl+Alt+Delete, 2. Windows and L to immediately lock the system, 3. Create a desktop icon to lock: right-click on the desktop, select create a shortcut, type "rundll32.exe user32.dll,LockWorkStation" without the quotation marks in the box for the location of the item, click next, name this shortcut as you wish, e.g., "Lock" - a desktop icon appears with that name, double click on it to instantly lock the computer device (Figures 1-4).

We re-audited after five months of the initial audit. We surveyed 100 computers and found 62 to be unlocked. We waited for three minutes at each computer and recorded data similar to the first audit as described above. All data was maintained in MS Excel format. GraphPad Prism (GraphPad Software, San Diego, USA) was used to do the statistical assessment and Fisher's Exact test was carried out. 

## Results

**Figure 5:**
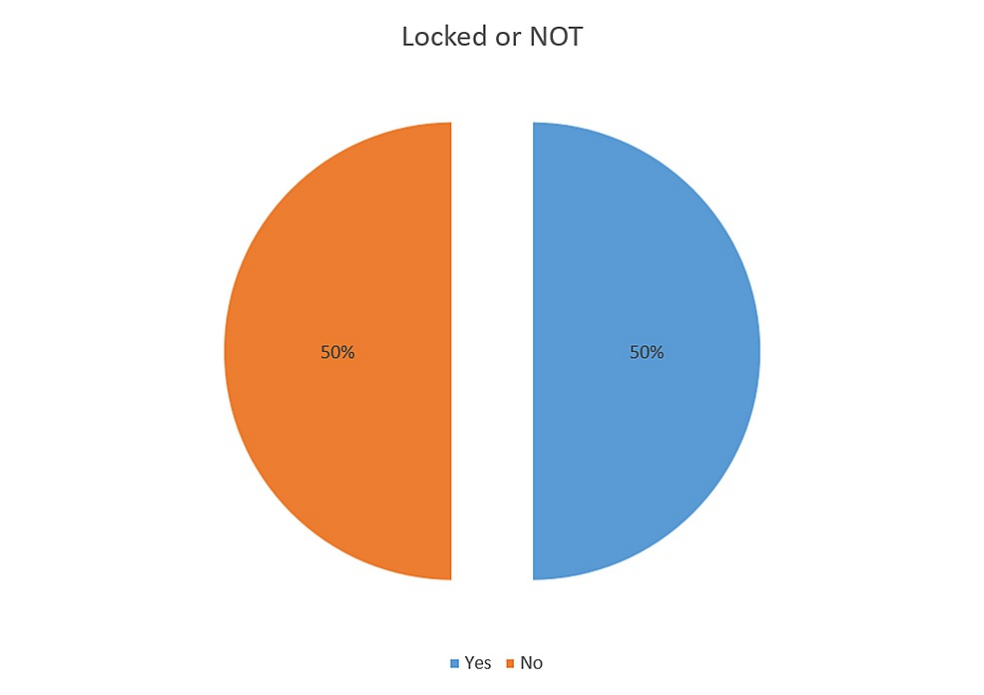
Results of Audit 1

**Figure 6:**
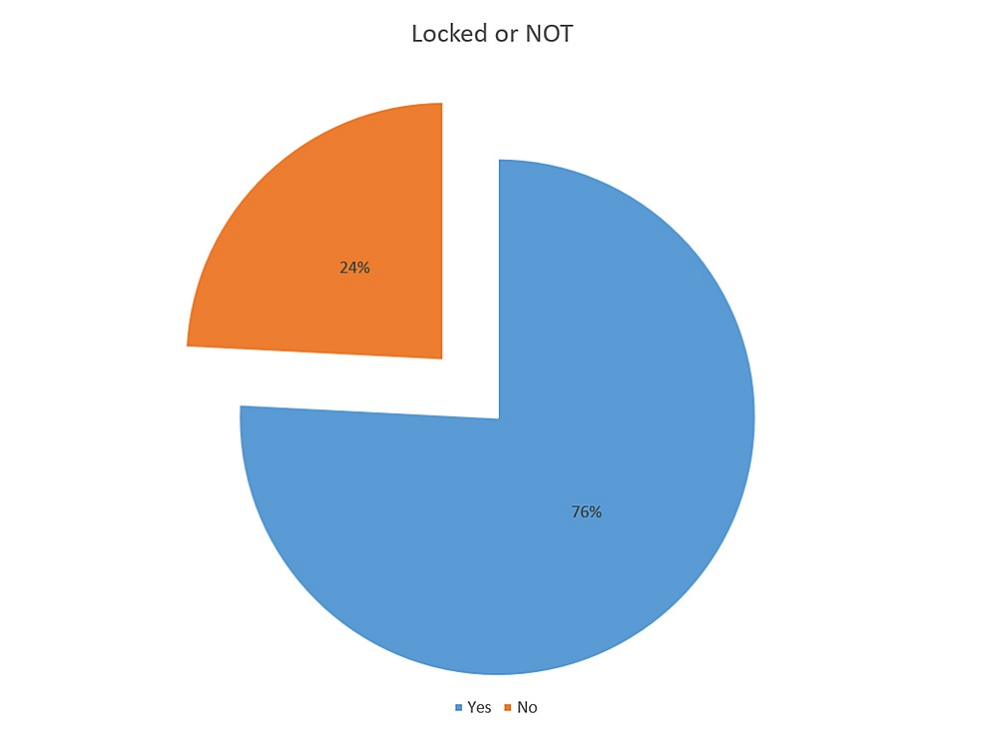
Results of Audit 2

Fisher's Exact test was done comparing the compliance pre- and post-intervention and the p-value was found to be 0.0142 (p<0.05). The results of the audits are presented in Table 1 and Figures 5-6.

**Table 1:** Results of audits IT: information technology

Parameter	Cycle 1	Cycle 2
Number of computers surveyed	58	109
Number of computers unoccupied	36	62
Number of computers found unlocked	18	15
Percentage of compliance with IT policy	50%	76%
Percentage of locked computers with more than one user logged on	22%	20%

## Discussion

The UK’s National Cyber Security Centre (NCSC) defines cyber security as how individuals and organisations reduce the risk of cyber-attack from malicious attempts to damage, disrupt, or gain unauthorised access to computer systems, networks or devices, via cyber means [2]. The IT Policy all across the United Kingdom National Health Service (NHS) Trusts clearly states, “When leaving desktop computers unattended they must be locked to prevent unauthorised access to documentation, services and systems.” 

Health care is one of the most targeted industries globally for cyber-attacks owing to the image of it being a ‘soft target’ due in part to staff behaviours [3-6]. There have been many instances that reveal our lack of “data safety” - for instance, the Wannacry attack. Recent research seems to suggest that staff do not show great awareness of the importance of data safety or logging out of computers. Up to 46% of health care breaches were found to be due to employee behaviour [1]. It is common knowledge that junior doctors are the backbone of any department - they have the most use of the computers, are the front-runners of health care in the wards and theatres, and hence use the computers most inside patient-accessible areas. Most of (90%) the reported patient harm incidents due to IT occurred at hospitals and only 10% occurred at Primary Care centres [1].

As of 28 May 2010, “Stolen data/hardware or Lost Data/hardware” tops the list of data breaches published by the UK Information Commissioner’s Office (ICO) in details of the 1007 data security breaches since late 2007. The NCSC in its guidelines mentions immediately locking a computer when not in use as a “Good Practice” [2]. This simple practice if not performed, can be disastrous. It can lead to a breach of patient confidentiality and a breach of Caldicott Principles. Loss of patient data can be a breach of the Data Protection Act and can lead to fines of up to half a million pounds. It can lead to patient harm [6] and harm to colleagues - for instance, using someone else’s account for requesting investigations and treatment. It can lead to prescribing errors [7]. It leaves a huge window open for data theft and installation of malware and ransomware. It also causes unnecessary delay in others logging in [8]. A lot of planning and money is being put into strengthening the cybersecurity of the NHS [2]. A complex interaction of machine-related, environment-related, and people-related factors occurs when an Electronic Health Care system is used [6-10], out of which human factors are very important as they cannot be addressed by funding alone [11].

We requested all junior doctors (FY to STR) via email in our Trust to come forward and consent for an interview about our study and 13 of them came forward. All the 13 had an IT Induction at least once with 38.5% having the induction more than a year ago. The IT Induction should be updated every year - a responsibility of the Trust and the IT Department that this training is delivered to the doctors on time. 23% of the doctors were not aware that it is a breach of Trust IT Policy if an unoccupied computer is unlocked and only 30% thought that it was important that they be compliant with this particular rule. This lack of awareness may partly be because NHS has historically been lax in its approach to cyber security. Recent data from 2018 suggests that only 12% of Trusts have achieved the mandatory objective of providing online training to their staff [3].

In our study, 61.5% found the current methods to lock computers to be easy. Struggle to find another computer, expecting to be back in a minute, fear of losing unsaved data and something more compelling turning up were the most common reasons that were commonly mentioned by junior doctors for not locking computers.

One of the junior doctors during the interview said, “Currently working in the surgical seminar room, if I locked my advice and left the room, I wouldn’t be able to come back to the computer and use it which is obviously not as much as an issue as patient data safety and confidentiality but it does mean sometimes you think twice about how to leave the computer. Have suggested Imprivata but nothing has been done about this.” Another junior doctor, while discussing options as to how they can be encouraged to lock the computer devices, said, "If there were more computers available specifically in the surgical seminar room I would be more inclined to lock all the time.”

The comment on the Struggle to find another computer brings us to the harsh fact that compared with other sectors, such as financial services, health care has chronically underinvested in IT infrastructure. Many NHS organisations spend as little as 1-2% of their annual budget on IT, compared with 4-10% in other sectors, with only a small proportion of that going on security [4, 5]. Certainly, increasing the number of computers would be helpful, but what kind of computers would we want to increase is the question. In our study, we found that the laptops on the trolleys that move around are the most prone to be left unlocked (38.88%).

“Imprivata would certainly be helpful,” said a colleague in his interview. Another colleague had a similar opinion and they said, “If there was Imprivata installed on every computer locking it would be really easy and quick but it's not the case and often it's a struggle just to find a computer. However, sometimes when I am using Imprivata it glitches and no matter how many times I tap off and try logging onto another computer it will not resolve. In those instances, the only thing I've found that helps is using a computer that doesn't have Imprivata installed.”

Imprivata (Imprivata, Lexington, USA) is a tap to log on - tap to log off card, which is user-specific and is a good option to encourage people to lock their computers. Yet in our study, we found 20% of unoccupied computers had smartcards still attached to them, which is not akin Imprivata but still is a card system for access.

A few junior doctors, while exploring the causes for not locking computers, mentioned, “Something more compelling turned up.” An article by G. Martin et al in 2017 states that “the culture of health care understandably focuses on caring for patients, even at the expense of security”. An example of this is staff sharing passwords [5]. We do agree that there could be something more compelling than locking a computer, but we have to agree that such situations will be very rare. The other reasons commonly cited were clearly due to lack of awareness and this awareness can only be increased by tailoring the IT Induction, delivering it promptly, and mandating that people remain updated with it.

However trivial it might seem, this little act of not locking a computer when leaving it unoccupied can lead to something as big as a hacking or ransomware attack, for instance, Wannacry [6,10,11]. We have all been through the scenario in IT Training where a “visitor” comes in and is able to access all information, leave pen drives loaded with malware, and is able to find many computers unlocked. Apart from this, we come across incidents where doctors have requested investigations for a patient from someone else’s account, or where one window was open for a particular patient and a wrong investigation was requested for them. This problem is not specific to one particular Trust or region. This audit was repeated in another Trust in the southern part of England and the compliance was found to be only 10%, although this particular trust still uses paper notes for the most part.

Via this paper, we want to emphasise the fact that cyber security is important and we as doctors can contribute hugely by doing simple things like locking our computers, which makes us compliant with the IT Usage Policy. We would want other trusts to follow our model and check their compliance. To improve compliance we suggest the “Educate-Communicate-Appreciate” model. IT Induction modules should stress upon this fact, include it in the Induction Multiple Choice Questions, test it at different exams like licensing exams, undergraduate exams, and other Royal College exams (Educate), display wallpapers with a message to lock computers, use posters, give feedback to each other (communicate) and do not ignore if you see a computer that is not locked (Appreciate).

For the purpose of Education, there is an excellent campaign by NHS Digital, which can be accessed at https://keepitconfidential.nhs.uk/campaign/. This website provides videos, posters, banners, quotes, etc that can be printed as posters and also shared digitally. The government has decided to spend millions in the forthcoming years to improve IT Security. However, this little step of not locking the computer cannot be helped with millions of pounds, if no intention from the end-user and if the causes for this problem are not addressed. Every ward has a specific number of patients and so the number of doctors required is known. Each doctor for the particular ward can be provided a hand- held mini device which they will use while they are working on that particular ward. These devices can then be deposited with the nurse-in-charge when leaving the ward. This we believe will increase accountability, reduce errors and make practice safer about Health IT. It will also solve the problem of people being afraid of not being able to find a vacant computer. Tap-to-log-in cards are another useful option to increase compliance.

## Conclusions

Not locking computers can lead to patient harm and financial losses. Compliance can be tested using our model. The suggested interventions can be applied to improve compliance. A lot is being done and much more needs to be done in the current era where the computer device has become an integral part of health care systems. It is important that health care professionals are aware of the advantages and shortcomings of the system they use and the necessity of good cyber ethics. Only then can we address seemingly simple problems at a basic level that might severely impact the whole system, especially the patients, in many ways. We hope this paper will make the reader reflect on their Data Security habits and build a safe, secure, and modern NHS.
